# The black esophagus and duodenum: a rare case report 

**Published:** 2020

**Authors:** Saad Saleem, Simcha Weissman, Sumair Ahmad

**Affiliations:** 1 *Mercy St. Vincent Medical Center, 2213 cherry street, Toledo, Ohio, USA *; 2 *Touro College of Osteopathic Medicine, New York, USA*; 3 *Department of Medicine, Mercy St. Vincent Medical Centre, Toledo, Ohio, USA*

**Keywords:** Esophagus, duodenum, ischemia

## Abstract

Acute esophageal necrosis is a rare esophageal disease, typically characterized by the circumferential black appearance of the esophagus mucosa that usually affects the distal esophagus. It commonly affects elderly men with multiple comorbidities. In the medical literature, some cases have been reported regarding acute necrotizing esophagus, but according to our knowledge, only one case has been reported concerning a patient with ischemic duodenum and esophagus. The case of a 71-year-old man with upper gastrointestinal bleeding and subsequent acute necrotizing esophagus and duodenum has been described below.

## Introduction

 Acute esophageal necrosis (AEN) is an extremely rare syndrome characterized by a circumferential black appearance of the esophagus mucosa that generally affects distal esophagus (1). It is also known as the black esophagus or acute necrotizing esophagitis. The etiology of acute esophagueal necrosis remains largely unclear since its first report by Golden Berg in 1990 (2). In the medical literature, there has been one case reported of AEN and black duodenum in a deceased patient secondary to diabetic ketoacidosis (3). In the present paper the clinical and endoscopic characteristics of a patient with a diagnosis of AEN and the black duodenum has been described. 

## Case Report

A 71 year old man with a medical history of alcohol abuse, diabetes mellitus, essential hypertension, and depression was admitted to the intensive care unit suffering from dyspnea, productive cough, hematemesis, and black colored stools. Physical examination revealed the patient in poor general condition; hypotensive with blood pressure 87/50 mm Hg, pulse 106 b.p.m, a temperature of 37.8 C and saturating 100% on 3 liters of oxygen. Laboratory tests showed hemoglobin 7.9 gram/deciliter (g/dl), leukocytes 7.9 kilo/microliter (k/uL), platelets 155 k/uL , sodium 129 mg/dL , potassium 3.4 millimole/liter (mmol/L) , BUN 22 mg/dL, creatinine 0.96 mg/dL, glucose 150 mg/dl, anion gap 25 mmol/L, lactic acid 5.3, ALT 44 units/liter (U/L), AST 74 U/L, total bilirubin 2.25 mg/dL, international normal ratio (INR) 1.6 and urine ketones negligible. 

Blood pressure responded to intravenous (IV) hydration 30ml/kg. The patient was started on IV Rocephin,and azithromycin for pneumonia based on symptoms and chest XR showing right lower lung infiltrates. The patient has suspected bleeding from esophageal varices, an indication of heavy alcohol abuse. He was started on proton pump inhibitors and an octreotide drip was administered. 

**Figure 1:**
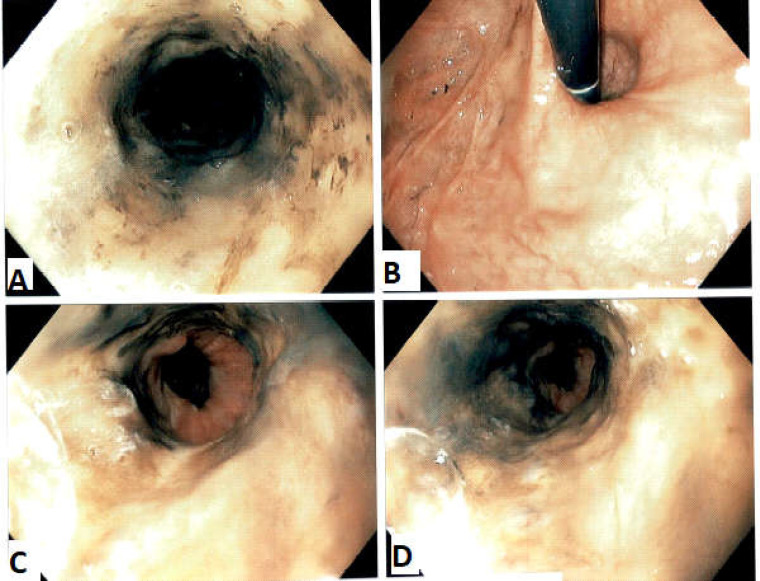
A, D showing circumferential, diffuse, black esophageal mucosa. C- Showing normal appearing esophagogastric junction. B- Mild erythema of the gastric fundus

Esophagogastroduodenoscopy (EGD) revealed circumferential, diffuse, friable mucosa extending from the proximal esophagus to the distal esophagus with sharp demarcation at the esophagogastric junction (GEJ) (Figure 1). 

**Figure 2:**
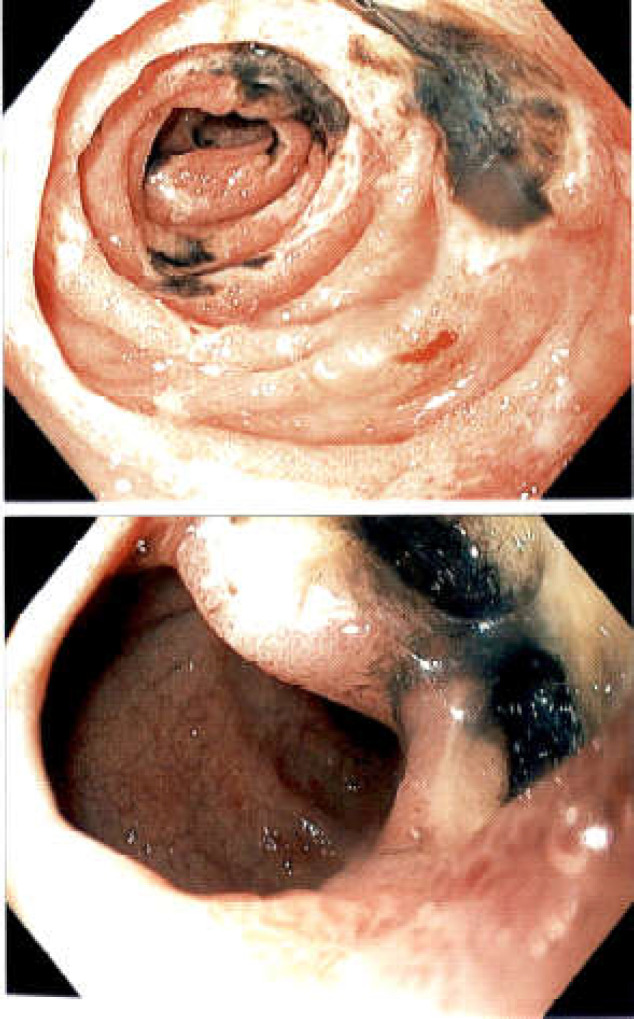
Showing multiple semi-lunar ulceration and black appearance in the duodenum

There were areas of black appearing esophageal mucosa in the distal esophagus. The gastric exam showed mild areas of erythema in the body but no Wischnewsky lesions, in other words gastric mucosal petechial hemorrhages. The duodenum revealed an area of semi-lunar ulceration in the bulb and several areas of ulceration/semi-circumferential with a black eschar-like appearance at the ulcerative base in the second and third part of the duodenum (Figure 2).

 Proton pump inhibitors continued, while octreotide drip was discontinued. Abdominal and pelvic CT with contrast was unremarkable for any thrombosis. It showed hepatic steatosis. The patient continued to improve on symptomatic management. One week later, a repeat EGD showed improving esophagitis and duodenum ulcers. Black discoloration of esophagus and duodenum was resolved. The patient continued to improve and was discharged. 

## Discussion

Acute esophageal necrosis is a rare but life-threatening condition with an estimated prevalence of up to 0.2 percent in autopsy series (4, 5). Gurvitis et al. described AEN as “poorly described in medical literature” (6). This condition is seen more in the elderly population likely due to underlying medical comorbidities including arteriosclerotic disease, diabetes mellitus, hypertension and pulmonary disease being more common (6). As noted in our patient, the vast majorities of patients are symptomatic and present with upper gastrointestinal bleeding in up to 90% of cases (6). 

It is a rare esophageal disorder with dark pigmentation of the esophagus found on upper endoscopy (6). This disorder is extremely rare as only 88 cases over the last 40 years have been reported in the medical literature (6).

In our case, the esophagitis was associated with multiple black necrotic ulcers in the duodenum, more severe in the second and third part of the duodenum. The pathogenesis of the acute esophageal necrosis remained unclear; it is postulated to occur due to severe topical esophageal mucosal injury from gastric acid and ischemia from low flow vascular state. AEN is frequently associated with a broad spectrum of antibiotic use, infections, gastric volvulus, diabetes, and binge alcohol use. 

Black discoloration occurs due to lipofuscin pigment, which gets accumulated due to ischemia. It commonly occurs in the distal esophagus at the gastroesophageal junction, likely due to being a watershed area and less vascularization in the distal esophagus (8, 9). Duodenum ischemia might occur due to gastric acid mucosal injury and low blood supply. Duodenum has a watershed area in the second part of the duodenum, where it receives dual blood supply from the most distal branches of the celiac artery and superior mesenteric artery branches. Watershed areas are particularly vulnerable to ischemia during systemic hypoperfusion as they are supplied by the most distal branches of the arteries. 

The diagnosis of AEN can only be made by upper endoscopy; a biopsy is not required (6). As illustrated in our case, circumferential black discoloration is usually seen with underlying friable tissue. With the increasing severity of the disease, the esophagus may be partially covered with thick, white exudates, which when dislodged will show underlying pink granulation tissue. 

Biopsy of the esophagus mucosa can be performed to rule out other conditions associated with darkened mucosa like melanosis, pseudomelanosis, melanoma, coal dust and pseudomembranous esophagitis. Histology usually shows necrosis of esophageal mucosa, submucosa and sometimes involvement of the adjacent muscle fibers. In our case histopathology could not be reported due to insufficient tissue sample, but repeat endoscopic evaluation showed resolution of black esophagus and duodenum. 

There is no specific treatment for AEN. Management usually involves supportive care with the resolution of endoscopic findings in most patients (10). It usually involves volume expansion with intravenous fluids and treatment of the underlying illness. Intravenous proton pump inhibitors should be started to suppress gastric acid production to reduce peptic acid injury to the esophagus and duodenum (11). The patient should be kept nil per oral for 24 hours to prevent esophageal perforation. Naso or orogastric tubes should be avoided due to the high risk of perforation from the necrotized esophageal wall. An esophagectomy can be considered in resistant cases (11). 

Mortality rates with AEN range from 13 to 35 percent usually occuring due to underlying diseases (7,10). AEN management requires the involvement of multidisciplinary care with an intensivist, gastroenterologist and surgeons. The underlying cause should be treated, and the patient should be managed in ICU. The prognosis of patients with AEN is based on an underlying medical condition. Local complications can include esophageal perforation and stricture.

## Conflict of interests

The authors declare that they have no conflict of interest.

## References

[1] MoretÃ M, Ojembarrena E, Zaballa M, Tánago JG, Ibánez S (1993). Idiopathic acute esophageal necrosis: not necessarily a terminal event. Endoscopy.

[2] Goldenberg SP, Wain SL, Marignani P (1990). Acute Necrotisizing Esophagitis. Gastroenterology.

[3] Garland J, Loper N, Philcox W, Ondruschka B, Kesha K, Stables S (2020). Black Duodenum in Fatal Diabetic Ketoacidosis. Am J Forensic Med Pathol.

[4] Etienne JP, Roge J, Delavierre P, Veyssier P (1969). Esophageal necrosis of vascular origin. Sem Hop.

[5] Postlethwait RW, Musser AW (1974). Changes in the esophagus in 1,000 autopsy specimens. J Thorac Cardiovasc Surg.

[6] Gurvits GE, Cherian K, Shami MN, Korabathina R, El-Nader EM, Rayapudi K (2015). Black esophagus: new insights and multicenter international experience in 2014. Dig Dis Sci.

[7] Lacy BE, Toor A, Bensen SP, Rothstein RI, Maheshwari Y (1999). Acute esophageal necrosis: report of two cases and a review of the literature. Gastrointest Endosc.

[8] Worrell SG, Oh DS, Greene CL, DeMeester SR, Hagen JA (2014). Acute esophageal necrosis: A cases series and long term follow-up. Ann Thorac Surg.

[9] Kimura Y, Seno H, Yamashita Y (2014). A case of acute necrotizing esophagitis. Gastrointestinal Endosc.

[10] Ben Soussan E, Savoye G, Hochain P, Hervé S, Antonietti M, Lemoine F (2002). Acute esophageal necrosis: a 1-year prospective study. Gastrointest Endosc.

[11] Carneiro M, Lescano M, Romanello L, Modena J, Carneiro F, Ramalho L (2005). Acute Esophageal Necrosis. Dig Endosc.

